# Once-daily single-inhaler versus twice-daily multiple-inhaler triple therapy in patients with COPD: lung function and health status results from two replicate randomized controlled trials

**DOI:** 10.1186/s12931-020-01360-w

**Published:** 2020-05-29

**Authors:** Gary T. Ferguson, Nicola Brown, Chris Compton, Thomas C. Corbridge, Kelly Dorais, Charles Fogarty, Catherine Harvey, Morrys C. Kaisermann, David A. Lipson, Neil Martin, Frank Sciurba, Marjorie Stiegler, Chang-Qing Zhu, David Bernstein

**Affiliations:** 1 Pulmonary Research Institute of Southeast Michigan, Farmington Hills, MI USA; 2 GlaxoSmithKline, Stockley Park, Uxbridge, Middlesex, UK; 3 GlaxoSmithKline, Brentford, Middlesex, UK; 4 GlaxoSmithKline, Research Triangle Park, NC, USA; 5 Northwestern University, Feinberg School of Medicine, Chicago, IL USA; 6 GlaxoSmithKline, Collegeville, PA USA; 7 Spartanburg Medical Research, Spartanburg, SC USA; 8 Perelman School of Medicine, University of Pennsylvania, Philadelphia, PA USA; 9 University of Leicester, Leicester, UK; 10 University of Pittsburgh Medical Center, Pittsburgh, PA USA; 11 University of North Carolina at Chapel Hill, Chapel Hill, NC USA; 12 Bernstein Clinical Research Center and Division of Immunology, Allergy and Rheumatology, University of Cincinnati College of Medicine, Cincinnati, OH USA

**Keywords:** Triple therapy, COPD, Lung function, Symptoms, Long-acting β_2_-agonist (LABA), Long-acting muscarinic antagonist (LAMA), Inhaled corticosteroid (ICS)

## Abstract

**Background:**

The comparative efficacy of inhaled corticosteroid/long-acting muscarinic antagonist/long-acting β_2_-agonist (ICS/LAMA/LABA) triple therapy administered via single or multiple inhalers in patients with chronic obstructive pulmonary disease (COPD) has not been evaluated comprehensively. We conducted two replicate trials comparing single- with multiple-inhaler ICS/LAMA/LABA combination in COPD.

**Methods:**

207608 and 207609 were Phase IV, 12-week, randomized, double-blind, triple-dummy non-inferiority trials comparing once-daily fluticasone furoate/umeclidinium/vilanterol (FF/UMEC/VI) 100/62.5/25 μg via Ellipta inhaler, with twice-daily budesonide/formoterol (BUD/FOR) 400/12 μg via metered-dose inhaler plus once-daily tiotropium (TIO) 18 μg via HandiHaler. Patients had symptomatic COPD and forced expiratory volume in 1 s (FEV_1_) < 50% predicted, or FEV_1_ < 80% predicted and ≥ 2 moderate or 1 severe exacerbations in the prior year. The primary endpoint in both trials was weighted mean change from baseline (wmCFB) in 0–24-h FEV_1_ at Week 12. Secondary endpoints included CFB in trough FEV_1_ at Day 84 and 85. Other endpoints included serial FEV_1_ and health status outcomes at Week 12. Safety was evaluated descriptively.

**Results:**

The modified per-protocol population included 720 and 711 patients in studies 207608 and 207609 (intent-to-treat population: 728 and 732). FF/UMEC/VI was non-inferior to BUD/FOR+TIO for wmCFB in 0–24-h FEV_1_ at Week 12 (Study 207608 treatment difference [95% confidence interval]: 15 mL [− 13, 43]; Study 207609: 11 mL [− 20, 41]). FF/UMEC/VI improved trough FEV_1_ CFB versus BUD/FOR+TIO at Day 84 and 85 (Day 85 treatment difference: Study 207608: 38 mL [10, 66]; Study 207609: 51 mL [21, 82]) and FEV_1_ at 12 and 24 h post-morning dose at Week 12 in both studies. No treatment differences were seen in health status outcomes. Safety profiles were similar between treatments; pneumonia occurred in 7 (< 1%) patients with FF/UMEC/VI and 9 (1%) patients with BUD/FOR+TIO, across both studies.

**Conclusions:**

FF/UMEC/VI was non-inferior to BUD/FOR+TIO for wmCFB in 0–24-h FEV_1_ at Week 12 in patients with COPD. Greater improvements in trough and serial FEV_1_ measurements at Week 12 with FF/UMEC/VI versus BUD/FOR+TIO, together with similar health status improvements and safety outcomes including the incidence of pneumonia, suggest that once-daily single-inhaler FF/UMEC/VI triple therapy is a viable option for patients looking to simplify their treatment regimen.

**Trial registration:**

GSK (207608/207609; NCT03478683/NCT03478696).

## Introduction

Chronic obstructive pulmonary disease (COPD) is a progressive disease, characterized by persistent respiratory symptoms, including dyspnea, cough, sputum production, and airflow limitation [1]. The 2020 Global Initiative for Chronic Obstructive Lung Disease (GOLD) guidelines recommend treatment initiation based on a patient’s symptoms and risk of exacerbations, with treatment escalation determined by the persistence of dyspnea and recurrent exacerbations while on treatment [1].

Escalation to triple therapy with an inhaled corticosteroid (ICS), a long-acting muscarinic antagonist (LAMA), and a long-acting β_2_-agonist (LABA) is recommended for patients who continue to experience clinically significant symptoms or recurrent exacerbations while receiving dual LAMA/LABA or ICS/LABA therapy [1]. Until recently, inhaled triple therapy has required the administration of multiple inhalers, several times a day, with the most commonly administered multiple-inhaler triple therapy in the USA being twice-daily budesonide/formoterol (BUD/FOR) plus once-daily tiotropium (TIO) [2]. The use of multiple-inhaler triple therapies has demonstrated improvements in lung function, health-related quality of life, hospitalization rates, and rescue medication use in patients with COPD compared with those receiving dual or monotherapies [3–12]. However, outside of the clinical trial environment, adherence to inhaled therapy for COPD is generally low, with non-adherence rates ranging from 50 to 80% [13–16], and patients with pulmonary diseases demonstrating lower adherence rates than many other chronic conditions [17]. This lack of adherence to medication can have a detrimental effect on patients’ well-being, and has been associated with increased risk of hospitalization and mortality, and reduced quality of life and productivity in patients with COPD [18].

Real-world evidence suggests that simplifying inhaler treatment regimens may improve adherence to and persistence with treatment for patients with COPD, potentially resulting in improved health outcomes due to a reduction in treatment discontinuation [18–20]. Although data are limited, some studies have suggested that simplifying treatment with a single inhaler may offer potential advantages in practicality and therapy adherence compared with multiple-inhaler therapy [19–21]. Recently, single inhalers containing ICS/LAMA/LABA have been developed. Phase III studies have demonstrated reductions in exacerbation rates, as well as improvements in health status and lung function, with single-inhaler triple therapy versus dual LAMA/LABA or ICS/LABA therapy, or LAMA monotherapy in patients with symptomatic COPD [12, 22–27]. The TRINITY study (NCT01911364) demonstrated that twice-daily single-inhaler triple therapy with beclometasone dipropionate/glycopyrronium bromide/formoterol fumarate had similar efficacy and safety as twice-daily beclometasone dipropionate/formoterol fumarate plus TIO multiple-inhaler triple therapy [27]. Nonetheless, further data comparing single-inhaler and multiple-inhaler therapy are needed.

Here, we report the results of two replicate Phase IV trials comparing the efficacy and safety of two ICS/LABA/LAMA combinations, one administered in a single inhaler (once-daily fluticasone furoate/umeclidinium/vilanterol [FF/UMEC/VI]) and one administered via multiple inhalers (twice-daily BUD/FOR plus once-daily TIO).

## Methods

### Study design

Study 207608 (NCT03478683) and Study 207609 (NCT03478696) were two replicate Phase IV, 12-week, randomized, double-blind, triple-dummy, parallel-group, multicenter, non-inferiority trials of single-inhaler triple therapy (FF/UMEC/VI) compared with multiple-inhaler triple combination therapy (BUD/FOR+TIO) in patients with COPD. Both trials were conducted from June 2018 to March 2019, with 59 centers in four countries included in Study 207608 and 58 centers in three countries included in Study 207609.

**Fig. 1:**
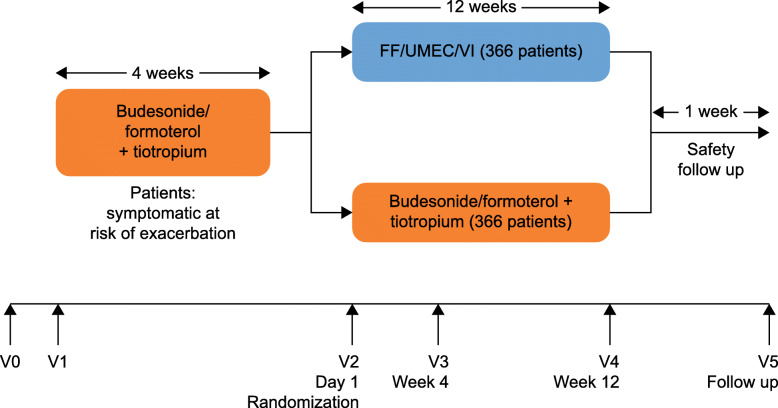
Study design. FF, fluticasone furoate; UMEC, umeclidinium; V, visit; VI, vilanterol.

Following screening (Visit 1) patients entered a 4-week run-in period during which they discontinued all existing COPD medications and received BUD/FOR (400/12 μg) twice daily via metered-dose inhaler (MDI) plus TIO (18 μg) once daily via HandiHaler (Boehringer Ingelheim International GmbH) plus placebo once daily via Ellipta dry-powder inhaler (GlaxoSmithKline). Following run-in (Visit 2; Day 1 of study), an Interactive Web Response System was used to randomize patients 1:1 to receive FF/UMEC/VI 100/62.5/25 μg once daily (in the morning) via Ellipta plus two inhalations of placebo twice daily via MDI (in the morning and evening) plus placebo once daily via HandiHaler (in the morning), or two inhalations of BUD/FOR 200/6 μg twice daily via MDI (in the morning and evening) plus TIO 18 μg once daily via HandiHaler (in the morning) plus placebo once daily (in the morning) via Ellipta for 84 days (Fig. 1). Patients self-administered their randomized treatment at home each day and during two on-treatment clinic visits at Week 4 (Visit 3) and Week 12 (Visit 4). A further safety follow-up telephone call or clinic visit (Visit 5) was conducted approximately 7 days after Visit 4 or end of study, whichever was first. Rescue albuterol was available as needed throughout the study but was withheld for at least 4 h prior to spirometry assessments.

All patients provided written informed consent. The trials were approved by the relevant ethics committee or institutional review board, in accordance with the International Council for Harmonisation of Technical Requirements for Registration of Pharmaceuticals for Human Use Good Clinical Practice and applicable country-specific requirements.

### Study population

At screening, eligible patients were outpatients ≥40 years of age, who were current or former smokers with a history of ≥ 10 pack-years with an established clinical history of COPD (as defined by the American Thoracic Society/European Respiratory Society [ATS/ERS] [28]). Patients were also required to have been receiving daily maintenance therapy for ≥ 3 months, have a post-bronchodilator forced expiratory volume in 1 s (FEV_1_) of < 50% predicted (or a post-bronchodilator FEV_1_ < 80% predicted and ≥ 2 moderate exacerbations or 1 severe exacerbation in the prior 12 months), a post-bronchodilator FEV_1_/forced vital capacity (FVC) ratio < 0.70 and a COPD Assessment Test (CAT) score ≥ 10.

Patients with a current diagnosis of asthma or other clinically significant respiratory disorders were excluded, as were those with pneumonia and/or a moderate or severe COPD exacerbation that had not resolved ≥ 14 days prior to screening and ≥ 30 days following the last dose of oral/systemic corticosteroid (if applicable), or a respiratory tract infection that had not resolved ≥ 7 days prior to screening. Patients at risk of non-compliance with study medication or attendance for scheduled visits, or unable to comply with the study procedures were also excluded. Compliance was continually assessed during the trial. Compliance between visits was assessed through querying the participant and recording the number of doses remaining in the Ellipta and MDI or the number of TIO or matching placebo capsules dispensed and taken by each participant and recorded in the electronic case report form (eCRF). Furthermore, during the 4-week run-in period patients were required to demonstrate 80–120% compliance with the run-in study medication, not experience any moderate or severe COPD exacerbations or pneumonia, and require no change in COPD medication. Full inclusion and exclusion criteria are provided in the Supplementary Materials.

### Study endpoints

The primary endpoint was the weighted mean change from baseline in FEV_1_ over 0–24 h at Week 12 (Day 84). Spirometry was conducted at each study visit, with ≥ 3 acceptable spirometry efforts obtained and the largest FEV_1_ measurement recorded. Secondary endpoints included the weighted mean change from baseline in FEV_1_ over 0–24 h on Day 1, and change from baseline in trough FEV_1_ on Days 2, 28, 84, and 85. Baseline was defined as the average of the two Day 1 pre-dose measurements. If one of the measurements was missing, the single remaining value was used as the baseline value. Other endpoints included the change from baseline in St George’s Respiratory Questionnaire (SGRQ) total score and CAT score at Week 12, and the proportion of responders based on the SGRQ total score (≥4-unit decrease from baseline) or CAT score (≥2-unit decrease from baseline) at Week 12.

The incidence of adverse events (AEs), serious AEs (SAEs), and AEs of special interest (AESIs) were recorded at each study visit. AESIs are events associated with the known class effects for ICS, LABA, and LAMA treatment, which allows for a comprehensive review of safety outcomes that is not restricted by specific preferred terms.

A pre-specified pooled analysis of both studies was performed for the efficacy and safety endpoints, which also included subgroup analyses by age (< 65, ≥ 65 years), percent predicted FEV_1_ at screening (≤ 30%, 30–< 50%, ≥ 50%), and baseline CAT score (< 20, ≥ 20).

### Statistical analyses

There were two analysis populations: the intent-to-treat (ITT) population included all randomized patients, except those randomized in error; the modified per protocol (mPP) population included all patients in the ITT population except those with a protocol deviation of not meeting the inclusion, exclusion, or randomization criteria.

Estimands allow for the evaluation of study objectives while taking into account the occurrence of intercurrent events (events that preclude the observation of an endpoint or affect its interpretation). In these studies, the primary estimand based on the mPP population excluded data following the occurrence of these intercurrent events: discontinuation of randomized study treatment; taking the wrong randomized study treatment; taking a prohibited medication; unblinding of randomized study treatment; treatment non-compliance; moderate/severe COPD exacerbation or pneumonia. Estimand based on the ITT population excluded data only following the discontinuation of randomized study treatment. Estimands were used for the analysis of all efficacy endpoints.

The sample size calculations used a one-sided 2.5% significance level and an estimate of residual standard deviation (SD) for 0–24-h weighted mean FEV_1_ of 230 mL. A study with 620 evaluable patients would have 90% power to determine non-inferiority of FF/UMEC/VI to BUD/FOR+TIO based on 0–24-h weighted mean FEV_1_ at Week 12, with a margin of non-inferiority of 50 mL, assuming a true mean treatment difference of 10 mL. Approximately 732 patients were planned to be randomized assuming 10% premature discontinuation of study treatment and 5% protocol deviation.

Non-inferiority for the primary endpoint of 0–24-h weighted mean FEV_1_ was analyzed in the mPP population using on-treatment data and a mixed model repeated measures (MMRM) analysis with covariates of baseline FEV_1_, visit, geographical region, treatment, visit by treatment, and visit by baseline interactions. If the lower bound of the two-sided 95% confidence interval (CI) around the (FF/UMEC/VI vs BUD/FOR+TIO) treatment difference was above − 50 mL then FF/UMEC/VI would be considered non-inferior to BUD/FOR+TIO. The primary endpoint was also analyzed in the ITT population. Only if non-inferiority was achieved with the primary analysis on the mPP population, would inference of superiority be made with the primary endpoint and other lung function endpoints on the ITT population. If superiority was not demonstrated for the primary endpoint, no inference would be drawn from *p*-values for treatment comparisons on secondary lung function endpoints in the ITT population. However, inference could still be drawn from *p*-values for treatment comparisons for other non-lung function endpoints (i.e. SGRQ and CAT) in the ITT population.

All other endpoints were assessed in the ITT population. The change from baseline in trough FEV_1_, serial FEV_1_, SGRQ total score, and CAT score was analyzed using a MMRM analysis with covariates of baseline value, geographical region, treatment, visit, visit by treatment, and visit by baseline interactions. The proportion of SGRQ total score and CAT score responders was analyzed using a generalized linear mixed model with a logit link function and covariates of treatment group, geographical region, visit, baseline, baseline by visit, and treatment by visit interactions. Safety endpoints were analyzed using descriptive statistics. The pre-specified pooled analysis of both studies was conducted using the pooled ITT population.

## Results

### Study population

**Fig. 2:**
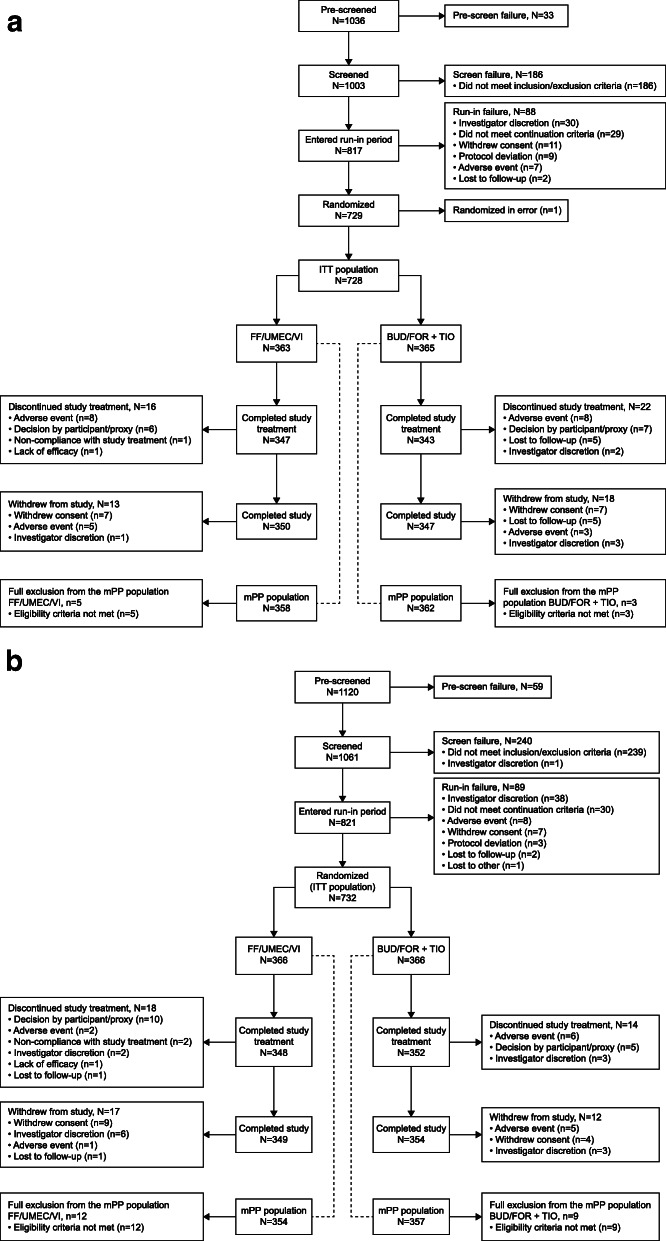
Patient disposition for (**a**) Study 207608 and (**b**) Study 207609 (ITT population). BUD, budesonide; FF, fluticasone furoate; FOR, formoterol; ITT, intent-to-treat; mPP, modified per protocol; TIO, tiotropium; UMEC, umeclidinium; VI, vilanterol.

The ITT population included 728 patients in Study 207608 (FF/UMEC/VI, *n* = 363; BUD/FOR+TIO, *n* = 365) (Fig. 2a), and 732 patients in Study 207609 (FF/UMEC/VI, *n* = 366; BUD/FOR+TIO, *n* = 366) (Fig. 2b). Of these, 720 patients in Study 207608 (FF/UMEC/VI, *n* = 358; BUD/FOR+TIO, *n* = 362) and 711 patients in Study 207609 (FF/UMEC/VI, *n* = 354; BUD/FOR+TIO, *n* = 357) were included in the mPP population. In total, 690 (95%) and 700 (96%) patients completed Study 207608 and Study 207609, respectively, with AEs or patient decision being the most common reasons for withdrawal (Fig. 2). Patient demographics and baseline characteristics were similar across treatment arms and studies (Table 1).

**Table 1:** Patient demographics and baseline characteristics (ITT population)

	Study 207608		Study 207609	
	FF/UMEC/VI *N* = 363	BUD/FOR + TIO *N* = 365	FF/UMEC/VI *N* = 366	BUD/FOR + TIO *N* = 366
**Age, years, mean (SD)**	65.4 (7.9)	64.9 (8.1)	65.5 (8.2)	65.1 (8.4)
**Female, n (%)**	180 (50)	164 (45)	180 (49)	179 (49)
**BMI, kg/m**^**2**^**, mean**	28.21 (6.58)	28.40 (6.93)	28.54 (7.56)	28.67 (7.14)
**Smoking history and status**	***n*** **= 363**	***n*** **= 365**	***n*** **= 366**	***n*** **= 366**
Current smoker, n (%)	186 (51)	168 (46)	170 (46)	190 (52)
Smoking pack-years, mean (SD)	47.9 (25.3)	47.9 (25.7)	48.3 (23.8)	50.2 (24.6)
**COPD type**	***n*** **= 356**	***n*** **= 358**	***n*** **= 362**	***n*** **= 363**
Chronic bronchitis, n (%)	139 (38)	121 (33)	138 (38)	146 (40)
Emphysema, n (%)	102 (28)	133 (36)	118 (32)	118 (32)
Chronic bronchitis and emphysema, n (%)	115 (32)	104 (28)	106 (29)	99 (27)
**COPD history**	***n*** **= 363**	***n*** **= 365**	***n*** **= 366**	***n*** **= 365**
Duration of COPD, years, mean (SD)	10.5 (6.8)	9.9 (6.8)	10.4 (7.3)	9.7 (7.0)
< 1 year, n (%)	2 (< 1)	4 (1)	5 (1)	4 (1)
≥ 1–< 5 years, n (%)	69 (19)	72 (20)	66 (18)	75 (20)
≥ 5–< 10 years, n (%)	123 (34)	133 (36)	126 (34)	152 (42)
≥ 10 years, n (%)	169 (47)	156 (43)	169 (46)	134 (37)
**Moderate COPD exacerbations in the previous 12 months, n (%)**	***n*** **= 363**	***n*** **= 365**	***n*** **= 366**	***n*** **= 366**
0	192 (53)	191 (52)	185 (51)	202 (55)
1	60 (17)	55 (15)	67 (18)	59 (16)
≥ 2	111 (31)	119 (33)	114 (31)	105 (29)
**Severe COPD exacerbations in the previous 12 months, n (%)**	***n*** **= 363**	***n*** **= 365**	***n*** **= 366**	***n*** **= 366**
0	324 (89)	325 (89)	326 (89)	310 (85)
1	34 (9)	35 (10)	37 (10)	47 (13)
≥ 2	5 (1)	5 (1)	3 (< 1)	9 (2)
**Screening lung function, mean (SD)**	***n*** **= 363**	***n*** **= 364**	***n*** **= 362**	***n*** **= 366**
Post-bronchodilator FEV_1_ (L)	1.176 (0.431)	1.199 (0.411)	1.128 (0.398)	1.181 (0.429)
Post-bronchodilator FEV_1_/FVC (% FEV_1_)	0.469 (0.110)	0.472 (0.115)	0.481 (0.107)	0.492 (0.109)
Post-bronchodilator FEV_1_, % predicted	42.5 (11.9)	42.3 (12.3)	41.4 (12.5)	42.8 (13.0)
Peak inspiratory flow rate, L/min*	208.37 (83.35)	200.68 (81.21)	191.04 (68.54)	195.91 (79.55)
**GOLD stage (% predicted FEV**_**1**_**), n (%)**	***n*** **= 363**	***n*** **= 364**	***n*** **= 362**	***n*** **= 366**
Stage I (≥80%)	0	0	0	0
Stage II (≥ 50 to < 80%)	76 (21)	82 (23)	71 (20)	84 (23)
Stage III (≥ 30 to < 50%)	236 (65)	226 (62)	219 (60)	221 (60)
Stage IV (< 30%)	51 (14)	56 (15)	72 (20)	61 (17)
**Reversibility to salbutamol**^**†**^**, n (%)**	***n*** **= 358**	***n*** **= 360**	***n*** **= 358**	***n*** **= 362**
Reversible	65 (18)	80 (22)	48 (13)	55 (15)
**CAT score at screening, mean (SD)**^**‡**^	21.6 (6.5)	22.0 (6.6)	22.2 (6.3)	22.3 (6.4)
**COPD medications at screening, n (%)**	***n*** **= 363**	***n*** **= 365**	***n*** **= 366**	***n*** **= 366**
ICS + LABA	121 (33)	137 (38)	123 (34)	115 (31)
ICS + LAMA + LABA	113 (31)	96 (26)	118 (32)	116 (32)
LABA + LAMA	55 (15)	42 (12)	59 (16)	67 (18)
LAMA	23 (6)	30 (8)	26 (7)	31 (8)

*207608, FF/UMEC/VI: *n* = 348; BUD/FOR+TIO: *n* = 354; 207609, FF/UMEC/VI: *n* = 342; BUD/FOR+TIO: *n* = 342. ^†^Reversible defined as an increase in FEV_1_ of ≥ 12% and ≥ 200 mL following administration of salbutamol. ^‡^207608, FF/UMEC/VI: *n* = 359; BUD/FOR+TIO: *n* = 364; 207609, FF/UMEC/VI: *n* = 364; BUD/FOR+TIO: *n* = 360. *BMI* body mass index; *BUD* budesonide; *CAT* COPD Assessment Test; *COPD* chronic obstructive pulmonary disorder; *FEV*_*1*_ forced expiratory volume in 1 s; *FF* fluticasone furoate; *FOR* formoterol; *FVC* forced vital capacity; *GOLD* Global Initiative for Chronic Obstructive Lung Disease; *ICS* inhaled corticosteroid; *ITT* intent-to-treat; *LABA* long-acting β_2_-agonist; *LAMA* long-acting muscarinic antagonist; *SD* standard deviation; *TIO* tiotropium; *UMEC* umeclidinium; *VI* vilanterol

### Efficacy

#### Primary endpoint

**Fig. 3:**
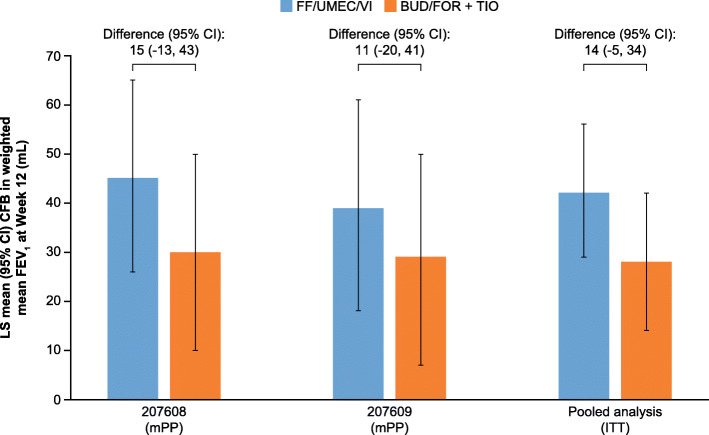
Change from baseline in weighted mean FEV_1_ over 0–24 h at Week 12. BUD, budesonide; CFB, change from baseline; CI, confidence interval; FEV_1_, forced expiratory volume in 1 s; FF, fluticasone furoate; FOR, formoterol; ITT, intent-to-treat; LS, least squares; mPP, modified per protocol; TIO, tiotropium; UMEC, umeclidinium; VI, vilanterol.

Once-daily FF/UMEC/VI demonstrated non-inferiority compared with twice-daily BUD/FOR plus once-daily TIO for the primary endpoint of weighted mean change from baseline in 0–24-h FEV_1_ at Week 12. Between-treatment differences (95% CI) in the mPP population were 15 mL (− 13, 43) in Study 207608 and 11 mL (− 20, 41) in Study 207609 (Table 2; Fig. 3). In the pooled analysis, the between-treatment difference was 14 mL (− 5, 34) in the ITT population (Table 2**;** Fig. 3).

**Table 2:** Weighted mean FEV_1_ 0–24 h at Week 12

	Study 207608 (mPP population)		Study 207609 (mPP population)		Pooled analysis (ITT population)	
	FF/UMEC/VI *N* = 358	BUD/FOR+TIO *N* = 362	FF/UMEC/VI *N* = 354	BUD/FOR+TIO *N* = 357	FF/UMEC/VI *N* = 729	BUD/FOR+TIO *N* = 731
n	282	272	274	277	674	665
LS mean (95% CI), mL	1210 (1191, 1230)	1195 (1175, 1215)	1185 (1163, 1206)	1174 (1153, 1195)	1199 (1185, 1213)	1185 (1171, 1198)
LS mean (SE) change from baseline, mL	45 (26, 65)	30 (10, 50)	39 (18, 61)	29 (7, 50)	42 (29, 56)	28 (14, 42)
Treatment difference (95% CI), mL	15 (−13, 43)		11 (−20, 41)		14 (−5, 34)	

*n* number of patients with analyzable data at the current time point

*BUD* budesonide; *CI* confidence interval; *FEV*_*1*_ forced expiratory volume in 1 s; *FF* fluticasone furoate; *FOR* formoterol; *ITT* intent-to-treat; *LS* least squares; *mPP* modified per protocol; *SE* standard error; *TIO* tiotropium; *UMEC* umeclidinium; *VI* vilanterol

#### Secondary endpoint

**Fig. 4:**
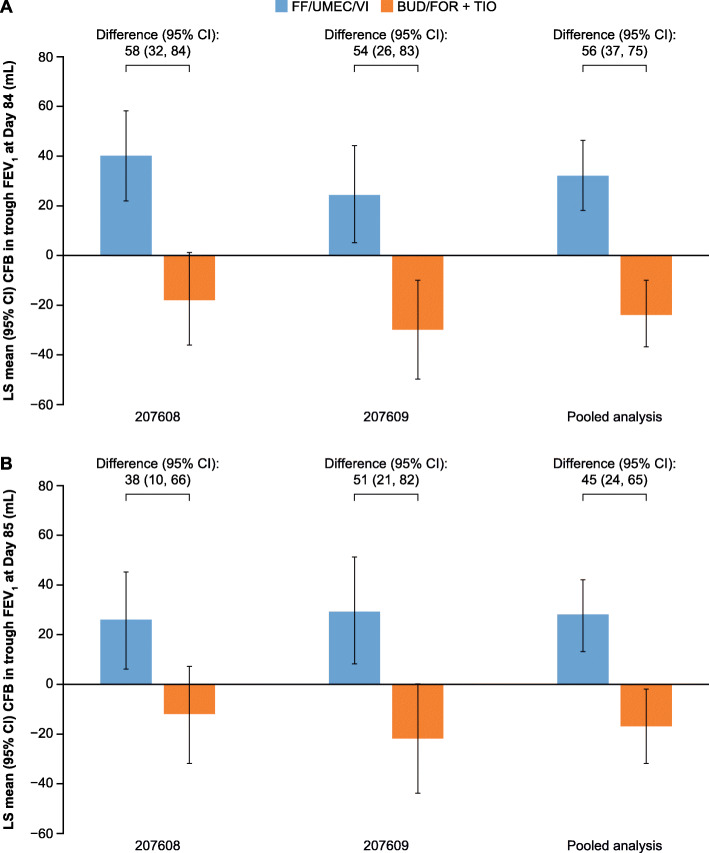
Change from baseline in trough FEV_1_ at (**a**) Day 84; (**b**) Day 85 (ITT population). BUD, budesonide; CFB, change from baseline; CI, confidence interval; FEV_1_, forced expiratory volume in 1 s; FF, fluticasone furoate; FOR, formoterol; ITT, intent-to-treat; LS, least squares; TIO, tiotropium; UMEC, umeclidinium; VI, vilanterol.

On Day 1, the weighted mean change from baseline in 0–24-h FEV_1_ was similar with FF/UMEC/VI and BUD/FOR+TIO in Study 207608 and 207609, and in the pooled analysis, in the ITT population (Table 3). On Day 84 and Day 85, change from baseline in trough FEV_1_ was numerically greater in patients receiving FF/UMEC/VI compared with BUD/FOR+TIO in Study 207608 and 207609, and in the pooled analysis, with the 95% CI for treatment difference excluding zero (Table 3; Fig. 4). This improvement with FF/UMEC/VI versus BUD/FOR+TIO was seen from Day 28 in Study 207608 and from Day 2 in Study 207609 (Table 3).

**Table 3:** Secondary lung function endpoints (ITT population)

Endpoint	Study 207608		Study 207609		Pooled analysis	
	FF/UMEC/VI *N* = 363	BUD/FOR+TIO *N* = 365	FF/UMEC/VI *N* = 366	BUD/FOR+TIO *N* = 366	FF/UMEC/VI *N* = 729	BUD/FOR+TIO *N* = 731
**0–24-h wmFEV**_**1**_**on Day 1**_**,**_**mL**						
n	354	355	360	356	714	711
LS mean (95% CI)	1217 (1202, 1233)	1226 (1211, 1242)	1195 (1182, 1209)	1191 (1178, 1205)	1206 (1196, 1216)	1209 (1199, 1219)
LS mean (95% CI) change from baseline	54 (39, 69)	63 (48, 78)	45 (32, 59)	41 (28, 55)	49 (39, 59)	52 (42, 62)
Treatment difference (95% CI)	−9 (−30, 13)		4 (−16, 23)		−3 (− 17, 11)	
**Trough FEV**_**1**_**on Day 2, mL**						
n	358	359	355	341	713	700
LS mean (95% CI)	1171 (1152,1190)	1181 (1161, 1200)	1164 (1147, 1180)	1138 (1121, 1155)	1167 (1154, 1180)	1160 (1147, 1173)
LS mean (95% CI) change from baseline	8 (−11, 27)	18 (−2, 37)	15 (−2, 32)	−10 (−28, 7)	11 (− 2, 24)	4 (−9, 17)
Treatment difference (95% CI)	− 10 (−37, 18)		26 (2, 49)		7 (− 11, 25)	
**Trough FEV**_**1**_**on Day 28, mL**						
n	355	353	353	354	708	707
LS mean (95% CI)	1210 (1192, 1227)	1148 (1131, 1165)	1193 (1174, 1211)	1130 (1111, 1149)	1201 (1188, 1214)	1139 (1127, 1152)
LS mean (95% CI) change from baseline	46 (29, 64)	−15 (−32, 2)	44 (25, 63)	−19 (− 37, 0)	45 (32, 58)	−16 (−29, −4)
Treatment difference (95% CI)	61 (37, 86)		63 (36, 89)		62 (44, 80)	
**Trough FEV**_**1**_**on Day 84, mL**						
n	344	340	346	343	690	683
LS mean (95% CI)	1203 (1185, 1221)	1145 (1127, 1164)	1173 (1153, 1193)	1119 (1099, 1139)	1188 (1174, 1201)	1132 (1118, 1146)
LS mean (95% CI) change from baseline	40 (22, 58)	−18 (−36, 1)	24 (5, 44)	−30 (−50, − 10)	32 (18, 46)	−24 (− 37, − 10)
Treatment difference (95% CI)	58 (32, 84)		54 (26, 83)		56 (37, 75)	
**Trough FEV**_**1**_**on Day 85, mL**						
n	341	337	343	342	684	679
LS mean (95% CI)	1189 (1169, 1208)	1151 (1131,1170)	1178 (1156, 1200)	1127 (1105, 1148)	1183 (1169, 1198)	1139 (1124, 1153)
LS mean (95% CI) change from baseline	26 (6, 45)	−12 (−32, 7)	29 (8, 51)	−22, (−44, 0)	28 (13, 42)	−17 (− 32, − 2)
Treatment difference (95% CI)	38 (10, 66)		51 (21, 82)		45 (24, 65)	

*n* number of patients with analyzable data at the current time point

*BUD* budesonide; *CI* confidence interval; *FEV*_*1*_forced expiratory volume in 1 s; *FF* fluticasone furoate; *FOR* formoterol; *ITT* intent-to-treat; *LS* least squares; *TIO* tiotropium; *UMEC* umeclidinium; *VI* vilanterol; *wm* weighted mean

#### Other endpoints

**Fig. 5:**
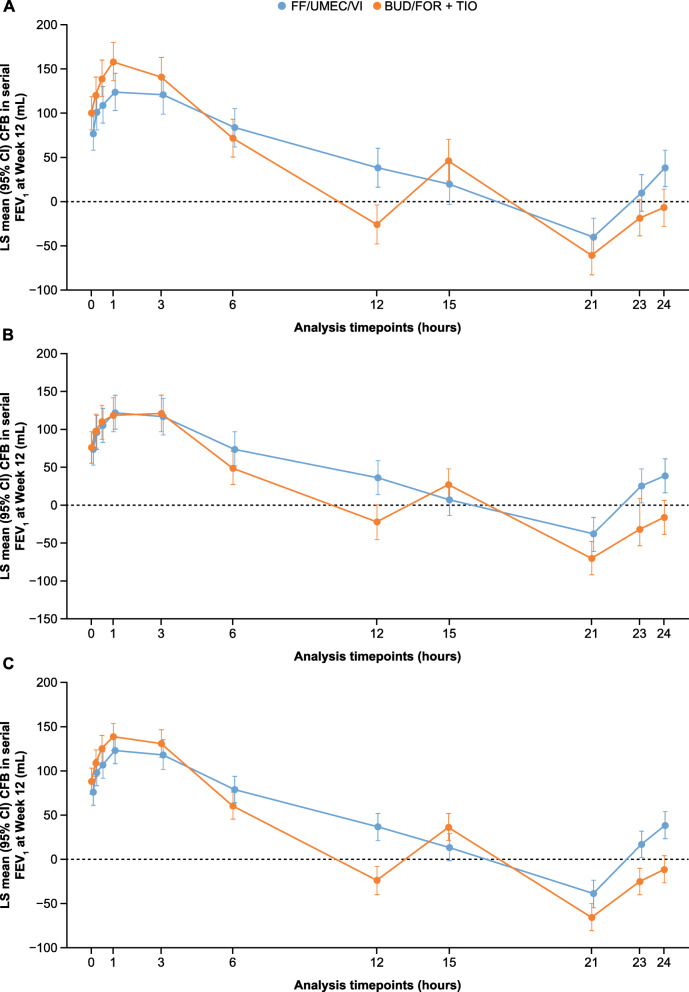
Change from baseline in serial FEV_1_ at Week 12 (ITT population). **a**. Study 207608; **b**. Study 207609; **c**. Pooled analysis. BUD, budesonide; CI, confidence interval; FEV_1_, forced expiratory volume in 1 s; FF, fluticasone furoate; FOR, formoterol; ITT, intent-to-treat; LS, least squares; TIO, tiotropium; UMEC, umeclidinium; VI, vilanterol.

In the pooled analysis, an improvement in change from baseline in serial FEV_1_ at Day 1 was seen with FF/UMEC/VI compared with BUD/FOR+TIO at the 12-h time point, while BUD/FOR+TIO demonstrated improvement over FF/UMEC/VI at the 15-h time point (i.e. after administration of the second dose), as seen in both individual studies (Supplementary Figure 1). By Week 12 the change from baseline in serial FEV_1_ in the pooled analysis was in favor of FF/UMEC/VI at the 12-, 21-, 23-, and 24-h time points, with 95% CIs for treatment difference excluding zero; similar results were seen in each study (Fig. 5). BUD/FOR+TIO demonstrated an improvement over FF/UMEC/VI at the 15-h time point (i.e. after administration of the second dose; Fig. 5).

In both studies and in the pooled analysis, no statistically significant between-treatment difference was seen in change from baseline in either SGRQ total score or CAT score or in the proportion of SGRQ total score responders or CAT score responders at Week 12 (Table 4, Supplementary Figures 2 and 3).

**Table 4:** Patient-reported outcome endpoints at Week 12 (ITT population)

Endpoint	Study 207608		Study 207609		Pooled analysis	
	FF/UMEC/VI *N* = 363	BUD/FOR+TIO *N* = 365	FF/UMEC/VI *N* = 366	BUD/FOR+TIO *N* = 366	FF/UMEC/VI *N* = 729	BUD/FOR+TIO *N* = 731
**SGRQ total score**						
n	344	342	343	342	687	684
LS mean (95% CI)	48.8 (47.8, 49.8)	48.7 (47.7, 49.7)	51.8 (50.7, 52.8)	51.7 (50.7, 52.8)	50.3 (49.5, 51.0)	50.2 (49.5, 51.0)
LS mean (95% CI) change from baseline	−1.2 (−2.2, −0.2)	− 1.3 (− 2.3, −0.3)	−1.5 (− 2.6, −0.4)	−1.5 (− 2.6, − 0.4)	−1.4 (− 2.1, − 0.6)	−1.4 (− 2.1, − 0.6)
Treatment difference (95% CI) *p*-value	0.1 (− 1.3, 1.5) 0.926		0.0 (− 1.5, 1.6) 0.974		0.0 (− 1.0, 1.1) 0.956	
n	345	343	343	344	688	687
Responders, n (%)	117 (34)	117 (34)	124 (36)	119 (35)	241 (35)	236 (34)
Odds ratio (95% CI) *p*-value	1.01 (0.74, 1.40) 0.928		1.09 (0.79, 1.50) 0.609		1.05 (0.84, 1.31) 0.671	
**CAT score**						
n	348	344	344	347	692	691
LS mean (95% CI)	19.8 (19.3, 20.3)	20.4 (19.8, 20.9)	21.1 (20.6, 21.6)	21.2 (20.7, 21.7)	20.4 (20.1, 20.8)	20.8 (20.4, 21.1)
LS mean (95% CI) change from baseline	−0.8 (−1.4, −0.3)	−0.2 (−0.8, 0.3)	− 0.2 (− 0.7, 0.3)	−0.1 (− 0.6, 0.4)	−0.5 (− 0.9, − 0.2)	−0.2 (− 0.6, 0.2)
Treatment difference (95% CI) *p*-value	− 0.6 (− 1.4, 0.2) 0.141		−0.1 (− 0.8, 0.6) 0.746		−0.3 (− 0.9, 0.2) 0.201	
n	349	344	344	348	693	692
Responders, n (%)	137 (39)	130 (38)	126 (37)	126 (36)	263 (38)	256 (37)
Odds ratio (95% CI) *p*-value	1.07 (0.78, 1.47) 0.655		1.02 (0.74, 1.40) 0.895		1.04 (0.83, 1.30) 0.732	

*BUD* budesonide; *CAT* COPD Assessment Test; *CI* confidence interval; *FF* fluticasone furoate; *FOR* formoterol; *ITT* intent-to-treat; *LS* least squares; *SGRQ* St George’s Respiratory Questionnaire; *TIO* tiotropium; *UMEC* umeclidinium; *VI* vilanterol

In the pooled analysis, a greater proportion of patients receiving BUD/FOR+TIO experienced at least one clinically important deterioration in trough FEV_1_ of ≥100 mL (BUD/FOR+TIO, 379/723 [52%] patients; FF/UMEC/VI, 277/726 [38%] patients).

#### Subgroup analysis

In the subgroup analyses of the pooled population by age, percent predicted FEV_1_ at screening or baseline CAT score, the results for weighted mean change from baseline in 0–24-h FEV_1_ at Week 12, and change in baseline in trough FEV_1_ at Day 84 and 85 were generally in line with the ITT population (Supplementary Figures 4 and 5). At Week 12, no treatment difference in the weighted mean change from baseline in 0–24-h FEV_1_ between FF/UMEC/VI and BUD/FOR+TIO was seen, with the exception of patients under 65 years of age (treatment difference: 31 mL) and patients with baseline CAT score ≥ 20 (29 mL) in favor of FF/UMEC/VI (Supplementary Figure 4). When analyzing change from baseline in trough FEV_1_ at Day 84, FF/UMEC/VI demonstrated a treatment benefit compared with BUD/FOR+TIO in all subgroups except in patients with a FEV_1_ ≤ 30% predicted at screening (Supplementary Figure 5A). A similar trend was seen at Day 85, although the treatment difference was generally less pronounced (Supplementary Figure 5B).

### Safety endpoints

Overall, the incidence of AEs, SAEs, and AESIs were similar across treatments and both Study 207608 and 207609, including the incidence of cardiovascular effects and pneumonia AESI (Table 5). In Study 207608, 5 (1%) and 6 (2%) patients reported pneumonia AESI in the FF/UMEC/VI and BUD/FOR+TIO groups, respectively, while 2 (< 1%) patients with FF/UMEC/VI and 3 (< 1%) with BUD/FOR+TIO reported pneumonia AESI in Study 207609.

**Table 5:** Overall safety outcomes (ITT population)

	Study 207608				Study 207609			
	FF/UMEC/VI *N* = 363		BUD/FOR+TIO *N* = 365		FF/UMEC/VI *N* = 366		BUD/FOR+TIO *N* = 366	
	n (%)	Rate [#]	n (%)	Rate [#]	n (%)	Rate [#]	n (%)	Rate [#]
**AEs**								
Any	131 (36)	2932.8 [244]	121 (33)	2651.8 [216]	92 (25)	2488.8 [205]	109 (30)	2321.2 [192]
Drug-related	23 (6)	372.6 [31]	16 (4)	233.3 [19]	9 (2)	182.1 [15]	10 (3)	145.1 [12]
Leading to permanent discontinuation or withdrawal	7 (2)	132.2 [11]	7 (2)	85.9 [7]	2 (< 1)	24.3 [2]	5 (1)	60.4 [5]
**SAEs**								
Any	25 (7)	444.7 [37]	14 (4)	221.0 [18]	12 (3)	279.2 [23]	17 (5)	266.0 [22]
Drug-related	4 (1)	60.1 [5]	0		1 (< 1)	24.3 [2]	1 (< 1)	12.1 [1]
Leading to permanent discontinuation or withdrawal	5 (1)	96.2 [8]	5 (1)	61.4 [5]	1 (< 1)	12.1 [1]	4 (1)	48.4 [4]
Fatal	0		0		0		1 (< 1)	12.1 [1]
**AESIs**								
Cardiovascular effects	10 (3)	132.2 [11]	8 (2)	135.0 [11]	11 (3)	157.8 [13]	8 (2)	120.9 [10]
Decreased BMD and associated fractures	5 (1)	72.1 [6]	3 (< 1)	36.8 [3]	2 (< 1)	36.4 [3]	4 (1)	48.4 [4]
LRTI excluding pneumonia	9 (2)	108.2 [9]	1 (< 1)	12.3 [1]	1 (< 1)	12.1 [1]	1 (< 1)	12.1 [1]
Pneumonia	5 (1)	60.1 [5]	6 (2)	73.7 [6]	2 (< 1)	24.3 [2]	3 (< 1)	60.4 [5]

Rate is the number of events per 1000 patient-year, calculated as the number of events × 1000 divided by the total treatment exposure

*AE* adverse event; *AESI* adverse event of special interest; *BMD* bone mineral density; *BUD* budesonide; *FF* fluticasone furoate; *FOR* formoterol; *ITT* intent-to-treat; *LRTI* lower respiratory tract infection; *SAE* serious adverse event; *TIO* tiotropium; *UMEC* umeclidinium; *VI* vilanterol

In Study 207608, a numerically higher number of patients in the FF/UMEC/VI group experienced lower respiratory tract infection (LRTI) excluding pneumonia AESI compared with patients in the BUD/FOR+TIO group (9 [2%] vs 1 [< 1%], respectively). This was not seen in Study 207609, where 1 (< 1%) and 1 (< 1%) patients reported LRTI excluding pneumonia AESI in the FF/UMEC/VI and BUD/FOR+TIO groups, respectively. No LRTI excluding pneumonia serious AESIs were reported in either study for either treatment arm.

No deaths were reported for patients receiving FF/UMEC/VI in either study, while 1 death was reported in the BUD/FOR+TIO group (lung adenocarcinoma) in Study 207609, not deemed to be related to study treatment.

## Discussion

Studies 207608 and 207609 both compared single-inhaler FF/UMEC/VI therapy against the most commonly prescribed multiple-inhaler triple therapy in the USA, BUD/FOR+TIO, and met the study-defined primary endpoint of non-inferiority for once-daily FF/UMEC/VI versus twice-daily BUD/FOR plus once-daily TIO for change from baseline in weighted mean FEV_1_ over 0–24 h at Week 12. No statistically significant differences between the treatments were seen in the change from baseline in SGRQ total score or CAT score at Week 12.

Of note, FF/UMEC/VI demonstrated improvements in the change from baseline in trough FEV_1_ at both Day 84 and 85 compared with BUD/FOR+TIO. Furthermore, results from the pooled serial FEV_1_ analysis indicate that FF/UMEC/VI was associated with greater improvements in lung function compared with BUD/FOR+TIO at specific time points within the 24 h (i.e. at both 12 and 24 h following the morning dose). These results suggest that, despite the perceived improvement after the second dose with twice-daily therapies [29], once-daily triple therapy with FF/UMEC/VI may reduce the variability of the treatment effect and thereby prevent the intermittent decline in lung function seen with twice-daily therapies [30, 31]. The greater improvements in FEV_1_ with FF/UMEC/VI at 12 h (i.e. prior to the second dose of BUD/FOR+TIO) and at 21, 23, and 24 h, suggest that patients who received BUD/FOR+TIO may be experiencing a loss of treatment effect followed by recovery to their baseline level following their additional required dose. This suggestion that FF/UMEC/VI may provide better protection against lung function deterioration is further supported by the lower proportion of patients experiencing a clinically important deterioration in trough FEV_1_ of ≥100 mL with FF/UMEC/VI compared with BUD/FOR+TIO.

Nighttime disturbances in patients with COPD are frequently reported, with over 65% of patients experiencing sleep disturbance owing to their symptoms [32, 33]. These nighttime disturbances are associated with patients having worse health status and worsening of COPD symptoms compared with patients who do not experience sleep disturbance [33]. Furthermore, it has also been reported that patients more frequently experience COPD symptoms that are worse than normal during the morning than any other time of day [34]. It is often thought that the administration of a second dose at night can alleviate these morning symptoms of COPD; however, the lack of difference in health status scores in this analysis suggest that there may be no difference between twice- and once-daily dosing for morning and nighttime symptom control. Nevertheless, the improvements seen in trough and serial FEV_1_ measures indicate that once-daily FF/UMEC/VI therapy may offer more consistent and sustained lung function benefits throughout the dosing interval than twice-daily BUD/FOR plus once-daily TIO.

Both treatment combinations were well tolerated, with low rates of treatment-related AEs or SAEs and few patients discontinuing owing to treatment-related AEs. A greater proportion of patients receiving FF/UMEC/VI experienced LRTI excluding pneumonia events compared with patients receiving BUD/FOR+TIO in Study 207608 and this higher incidence was driven primarily by bronchitis events (7 [2%] vs 1 [< 1%] of patients in FF/UMEC/VI and BUD/FOR+TIO arms, respectively). Of note, a greater proportion of patients with chronic bronchitis were enrolled to the FF/UMEC/VI arm compared with the BUD/FOR+TIO arm in Study 207608, whereas an equal proportion of patients with chronic bronchitis were enrolled to both treatment arms in Study 207609, where no difference in the incidence of LRTI excluding pneumonia events was observed. It is important to note that the incidence of cardiovascular or pneumonia AESIs, which have both been associated with these medication classes [35–38], were low and similar with both treatments and in both studies.

One limitation of these studies was the short period of assessment (12 weeks), which restricts any analysis of long-term safety outcomes and treatment effects (e.g. incidence of exacerbation events between the treatment combinations).

It is interesting to note that a greater treatment difference in trough FEV_1_ between FF/UMEC/VI and BUD/FOR+TIO was seen on Day 84 compared with Day 85. As treatment on Day 84 was self-administered at home, while on Day 85 it was administered in the clinic, this may suggest that some patients do not self-administer their second daily treatment dose at the optimal time, potentially reducing the effect of the treatment. Adherence is defined as not just conforming to the recommendations regarding the dosage and frequency of medication administration but also the timing of the administration [39]. Simplifying treatment regimens with a once-daily therapy has been suggested to improve adherence to therapy with respect to the timing of the medication compared with twice-daily therapy, which in turn may lead to improved clinical outcomes [19–21]. However, dummy inhalers were used in these studies to ensure blinding and, as such, the studies were not designed to assess the impact of single or multiple inhalers nor once-daily or twice-daily therapy on adherence. Furthermore, the implementation of randomization criteria, to ensure that patients were ≥ 80 to ≤ 120% compliant with the run-in study medication, maximized the adherence of the patient population and may therefore not be reflective of adherence in the general COPD population. It can reasonably be expected that the real-world treatment effect of these medications would reflect any impact of the dosing regimens on adherence, and future real-world or open-label studies to determine the impact of patient’s adherence on treatment effectiveness may, therefore, reveal greater differences between treatments.

## Conclusion

Once-daily single-inhaler triple therapy with FF/UMEC/VI provides similar overall improvements in weighted mean FEV_1_ and health status, and a similar safety profile, including a low incidence of pneumonia, as twice-daily multiple-inhaler triple therapy with BUD/FOR+TIO. These results suggest that FF/UMEC/VI is a viable treatment option for patients who wish to simplify their treatment regimen from multiple- to single-inhaler triple therapy. The greater improvements with FF/UMEC/VI over BUD/FOR+TIO in trough FEV_1_ at 12 and 24 h further suggest that once-daily FF/UMEC/VI therapy may offer more consistent and sustained lung function benefits throughout the dosing interval compared with twice-daily BUD/FOR + once-daily TIO therapy.

## Supplementary information

**Additional file 1.** Full inclusion and exclusion criteria and supplementary figures 1–5.

## Data Availability

Anonymized individual participant data and study documents can be requested for further research from www.clinicalstudydatarequest.com.

## Abbreviations

AE: Adverse event
AESI: Adverse event of special interest
ATS: American Thoracic Society
BMI: Body mass index
BUD: Budesonide
CAT: COPD Assessment Test
CFB: Change from baseline
CI: Confidence interval
COPD: Chronic obstructive pulmonary disease
ERS: European Respiratory Society
FEV_1_: Forced expiratory volume in 1 s
FF: Fluticasone furoate
FOR: Formoterol
FVC: Forced vital capacity
GOLD: Global Initiative for Chronic Obstructive Lung Disease
ICS: Inhaled corticosteroid
ITT: Intent-to-treat
LABA: Long-acting β_2_-agonist
LAMA: Long-acting muscarinic antagonist
LRTI: Lower respiratory tract infection
MDI: Metered-dose inhaler
MMRM: Mixed measures repeated model
mPP: Modified per protocol
SAE: Serious adverse event
SD: Standard deviation
SGRQ: St George’s Respiratory Questionnaire
TIO: Tiotropium
UMEC: Umeclidinium
VI: Vilanterol
wmCFB: Weighted mean change from baseline
