# Supplementary material for: Once-daily single-inhaler versus twice-daily multiple-inhaler triple therapy in patients with COPD: lung function and health status results from two replicate randomized controlled trials
Source: Respir Res. 2020 May 29;21:131. doi: 10.1186/s12931-020-01360-w (PMC7257245; doi:10.1186/s12931-020-01360-w)
Supplement: Supplementary file 1 — Additional file 1. Full inclusion and exclusion criteria and supplementary figures 1–5. [file 12931_2020_1360_MOESM1_ESM.docx]

**Additional File 1**

**Inclusion and exclusion criteria for Studies 207608 and 207609**

**Inclusion Criteria**

Participants are eligible to be included in the study only if all of the following criteria apply:

1. Informed consent: capable of giving signed informed consent prior to study start which includes compliance with the requirements and restrictions listed in the informed consent form and in this protocol.
2. Type of participant: Outpatient.
3. Age: Participants 40 years of age or older at screening (Visit 1).
4. Gender: Male or female participants.
   Female participants: A female participant is eligible to participate if she is not pregnant, not breastfeeding, and at least one of the following conditions applies:
   1. Not a woman of childbearing potential (WOCBP)
      OR
   2. A WOCBP who agrees to follow the contraceptive guidance in Appendix 3 during the treatment period and until the safety follow-up contact after the last dose of study treatment.
5. COPD Diagnosis: An established clinical history of COPD in accordance with the definition by the American Thoracic Society/European Respiratory Society [1].
6. Smoking History: Current or former cigarette smokers with a history of cigarette smoking of ≥10 pack-years at screening (Visit 1) (number of pack years = [number of cigarettes per day/20] x number of years smoked [e.g. 20 cigarettes per day for 10 years, or 10 cigarettes per day for 20 years]). Previous smokers are defined as those who have stopped smoking for at least 6 months prior to Visit 1. Note: Pipe and/or cigar use cannot be used to calculate pack-year history.
7. Severity of COPD symptoms: A score of ≥10 on the COPD Assessment Test (CAT) at screening (Visit 1).
8. Severity of Disease: Participants must demonstrate at screening: a post-bronchodilator FEV_1_ <50% predicted normal OR a post-bronchodilator FEV_1_ <80% predicted normal and a documented history of ≥2 moderate exacerbations or one severe (hospitalized) exacerbation in the previous 12 months. Participants must also have a measured post albuterol/salbutamol FEV_1_/forced vital capacity (FVC) ratio of <0.70 at screening. (Note: Percent predicted will be calculated using the European Respiratory Society Global Lung Function Initiative reference equations [2]; Note: A documented history of a COPD exacerbation (e.g. medical record verification) is a medical record of worsening COPD symptoms that required systemic/oral corticosteroids and/or antibiotics (for a moderate exacerbation) or hospitalization (for a severe exacerbation). Prior use of antibiotics alone does not qualify as an exacerbation history unless the use was associated with treatment of worsening symptoms of COPD, such as increased dyspnea, sputum volume, or sputum purulence (color). Participant verbal reports are not acceptable.)
9. Existing COPD maintenance treatment: participant must have been receiving daily maintenance treatment for their COPD for at least 3 months prior to screening (Note: Participants taking only as-needed COPD medications are not eligible).

**Exclusion Criteria**

Participants are excluded from the study if any of the following criteria apply:

1. Pregnancy: Women who are pregnant or lactating or are planning on becoming pregnant during the study.
2. Asthma: Participants with a current diagnosis of asthma. (Participants with a prior history of asthma are eligible if they have a current diagnosis of COPD).
3. α1-antitrypsin deficiency: Participants with α1-antitrypsin deficiency as the underlying cause of COPD.
4. Other respiratory disorders: Participants with active tuberculosis, lung cancer, and clinically significant: bronchiectasis, sarcoidosis, lung fibrosis, pulmonary hypertension, interstitial lung disease or other active pulmonary diseases.
5. Lung resection: Participants who have undergone lung volume reduction surgery within the 12 months prior to screening.
6. Risk Factors for Pneumonia: immune suppression (e.g. advanced human immunodeficiency virus with high viral load and low CD4 count, lupus on immunosuppressants) that in the opinion of the investigator would increase risk of pneumonia or other risk factors for pneumonia (e.g. neurological disorders affecting control of the upper airway, such as Parkinson’s Disease, Myasthenia Gravis). Participants at potentially high risk for pneumonia (e.g. very low body mass index [BMI], severely malnourished, or very low FEV_1_) will only be included at the discretion of the Investigator.
7. Pneumonia and/or moderate or severe COPD exacerbation that has not resolved at least 14 days prior to screening and at least 30 days following the last dose of oral/systemic corticosteroids (if applicable).
8. Respiratory tract infection that has not resolved at least 7 days prior to screening.
9. Abnormal Chest x-ray: Chest x-ray (posteroanterior and lateral) reveals evidence of pneumonia or a clinically significant abnormality not believed to be due to the presence of COPD, or another condition that would hinder the ability to detect an infiltrate on chest X-ray (e.g. significant cardiomegaly, pleural effusion or scarring). All participants will have a chest X-ray at screening Visit 1 (or historical radiograph or computerized tomography [CT] scan obtained within 3 months prior to screening). Note: Participants who have experienced pneumonia and/or moderate or severe COPD exacerbations within 3 months of screening must provide a post pneumonia/exacerbation chest X-ray or have a chest X-ray conducted at screening. For sites in Germany: If a chest x-ray (or CT scan) within 3 months prior to screening (Visit 1) is not available, approval to conduct a diagnostic chest x-ray will need to be obtained from the Federal Office for Radiation Protection (BfS).
10. Other diseases/abnormalities: Participants with historical or current evidence of clinically significant cardiovascular, neurological, psychiatric, renal, hepatic, immunological, gastrointestinal, urogenital, nervous system, musculoskeletal, skin, sensory, endocrine (including uncontrolled diabetes or thyroid disease) or hematological abnormalities that are uncontrolled. Significant is defined as any disease that, in the opinion of the Investigator, would put the safety of the participant at risk through participation, or which would affect the efficacy or safety analysis if the disease/condition exacerbated during the study.
11. Unstable liver disease: ALT >2x upper limit of normal (ULN); and bilirubin >1.5x ULN (isolated bilirubin >1.5x ULN is acceptable if bilirubin is fractionated and direct bilirubin <35%). Current active liver or biliary disease (with the exception of Gilbert’s syndrome or asymptomatic gallstones or otherwise stable chronic liver disease per investigator assessment). Notes: Stable chronic liver disease should generally be defined by the absence of ascites, encephalopathy, coagulopathy, hypoalbuminemia, esophageal or gastric varices, or persistent jaundice, or cirrhosis. Chronic stable hepatitis B and C (e.g. presence of hepatitis B surface antigen (HBsAg) or positive hepatitis C antibody test result at screening or within 3 months prior to first dose of study treatment) are acceptable if participant otherwise meets entry criteria.
12. Unstable or life threatening cardiac disease: Participants with any of the following at screening (Visit 1) would be excluded:
    1. Myocardial infarction or unstable angina in the last 6 months
    2. Unstable or life threatening cardiac arrhythmia requiring intervention in the last 3 months
    3. New York Heart Association Class IV Heart failure.
13. Abnormal and clinically significant 12-lead ECG finding at Visit 1. The Investigator will determine the clinical significance of each abnormal ECG finding in relation to the participant’s medical history and exclude participants who would be at undue risk by participating in the trial. An abnormal and clinically significant finding that would preclude a participant from entering the trial is defined as a 12-lead ECG tracing that is interpreted at, but not limited to, any of the following:
    1. Atrial fibrillation (AF) with rapid ventricular rate >120 beats per minute
    2. Sustained and non-sustained ventricular tachycardia
    3. Second degree heart block Mobitz type II and third degree heart block (unless pacemaker or defibrillator had been inserted)
    4. QT interval corrected for heart rate by ≥500 msec in participants with QRS <120 msec and QTcF ≥530 msec in participants with QRS ≥120 msec.
14. Contraindications: A history of allergy or hypersensitivity to any corticosteroid, anticholinergic/muscarinic receptor antagonist, β_2_-agonist, lactose/milk protein or magnesium stearate or a medical condition such as narrow-angle glaucoma, prostatic hypertrophy or bladder neck obstruction that, in the opinion of the Investigator, contraindicates study participation.
15. Cancer: Participants with carcinoma that has not been in complete remission for at least 3 years. Participants who have had carcinoma in situ of the cervix, squamous cell carcinoma and basal cell carcinoma of the skin would not be excluded based on the 3 year waiting period if the participant has been considered cured by treatment.
16. Oxygen therapy: Use of long-term oxygen therapy described as resting oxygen therapy >3 L/min at screening (Oxygen use ≤3 L/min flow is not exclusionary.)
17. Medication prior to spirometry: Participants who are medically unable to withhold their albuterol/salbutamol for the 4-hour period required prior to spirometry testing at each study visit.
18. Pulmonary rehabilitation: Participants who have participated in the acute phase of a pulmonary rehabilitation program within 4 weeks prior to screening or participants who plan to enter the acute phase of a pulmonary rehabilitation program during the study. Participants who are in the maintenance phase of a pulmonary rehabilitation program are not excluded.
19. Drug/alcohol abuse: Participants with a known or suspected history of alcohol or drug abuse within the last 2 years.
20. Non-compliance: Participants at risk of non-compliance, or unable to comply with the study procedures. Any infirmity, disability, or geographic location that would limit compliance for scheduled visits.
21. Questionable validity of consent: Participants with a history of psychiatric disease, intellectual deficiency, poor motivation or other conditions that will limit the validity of informed consent to participate in the study.
22. Affiliation with Investigator site: study Investigators, sub-Investigators, study coordinators, employees of a participating Investigator or study site, or immediate family members of the aforementioned that is involved with this study.
23. Inability to read: In the opinion of the Investigator, any participant who is unable to read and/or would not be able to complete study related materials.
24. Medication prior to screening: Use of the following medications within the following time intervals prior to Visit 1

| **Medication** | **No use within the following time intervals prior to screening** |
| --- | --- |
| Inhaled short-acting anticholinergics | 6 hours |
| Inhaled short-acting β_2_-agonists* | ≥4 hours |
| Inhaled short-acting anticholinergics + short-acting β_2_-agonist combination | 6 hours |
| Long-term antibiotic therapy | Participants receiving antibiotics for long-term therapy (≥30 days) are not eligible for the study.  (Antibiotics are allowed for the short-term treatment (≤14 days) of an exacerbation or for short-term treatment (≤14 days) of other acute infections during the study) |
| Systemic, oral, parenteral corticosteroids | 30 days (During the study oral/systemic corticosteroids may be used for ≤14 days to treat COPD exacerbations/pneumonia)  Intra-articular injections are allowed |
| Any other investigational drug | 30 days or 5 half-lives, whichever is longer |

*Rescue albuterol/salbutamol will be provided and is permitted during the study.

**Randomization Criteria**

At the end of the run-in period (Visit 2), study participants must fulfil the following additional criteria in order to be randomized into the study and enter the treatment period:

1. Compliance with run-in study medication. Compliance with each run-in study medication will be assessed by the Investigator, any participant <80% or >120% compliant with any of the three inhalers (Ellipta, HandiHaler, or MDI) will be excluded.
2. COPD exacerbation or pneumonia. Participants that experience a moderate or a severe COPD exacerbation or pneumonia during the run-in period will be excluded.
3. Changes in COPD medication. Any participant that requires any change in COPD medication during the run-in period will be excluded. This includes a temporary change in COPD medication.

**References**

1. Celli BR, MacNee W, ATS/ERS Task Force. Standards for the diagnosis and treatment of patients with COPD: A summary of the ATS/ERS position paper. Eur Respir J 2004; 23: 932-946.
2. Quanjer P, Stanojevic S, Cole T, Baur X, Hall G, Culver B, et al. Multi-ethnic reference values for spirometry for the 3-95-yr age range: The global lung function 2012 equations. Eur Respir J 2012;, 40: 1324-1343.

**Supplementary Figure 1.** Least squares mean change from baseline in serial FEV_1_ at Day 1 (ITT population).


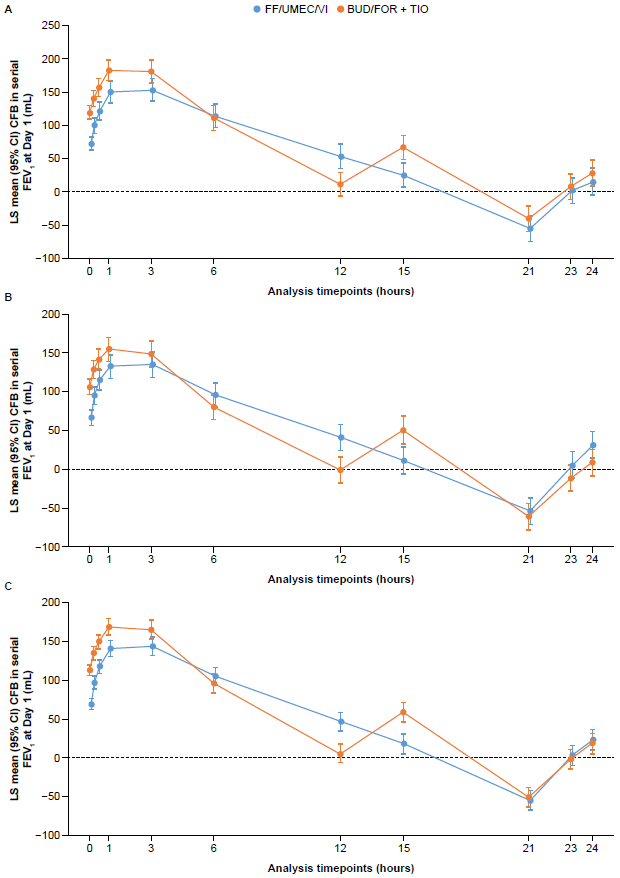


A. Study 207608; B. Study 207609; C. Pooled analysis.
BUD, budesonide; CFB, change from baseline; CI, confidence interval; FEV_1_, forced expiratory volume in 1 second; FF, fluticasone furoate; FOR, formoterol; ITT, intent-to-treat; LS, least squares; TIO, tiotropium; UMEC, umeclidinium; VI, vilanterol.

**Supplementary Figure 2.** Change from baseline in SGRQ total score at Week 12 (hypothetical estimand).


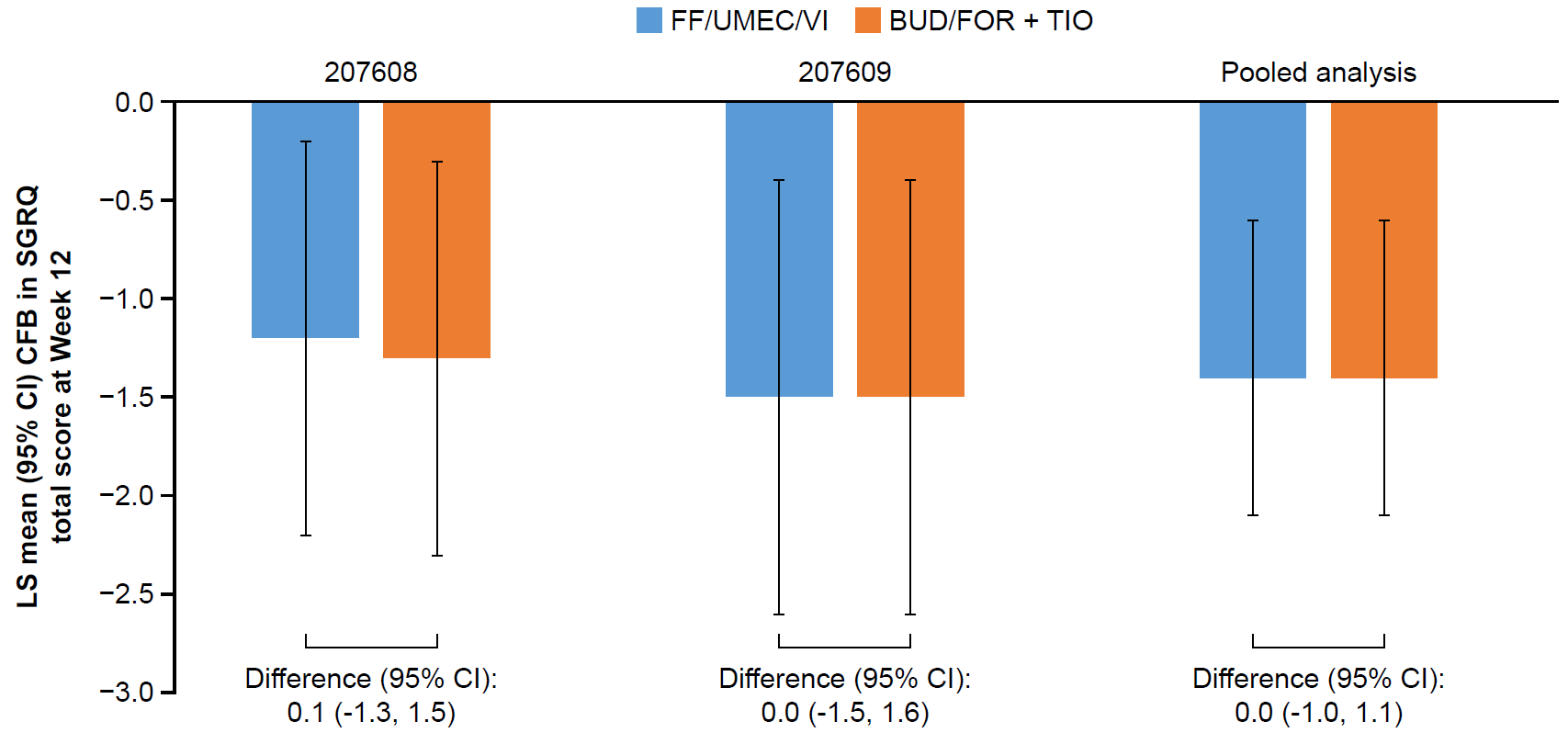


BUD, budesonide; CI, confidence interval; FF, fluticasone furoate; FOR, formoterol; LS, least squares; SGRQ, St George’s Respiratory Questionnaire; TIO, tiotropium; UMEC, umeclidinium; VI, vilanterol.

**Supplementary Figure 3.** Change from baseline in CAT score at Week 12 (hypothetical estimand).


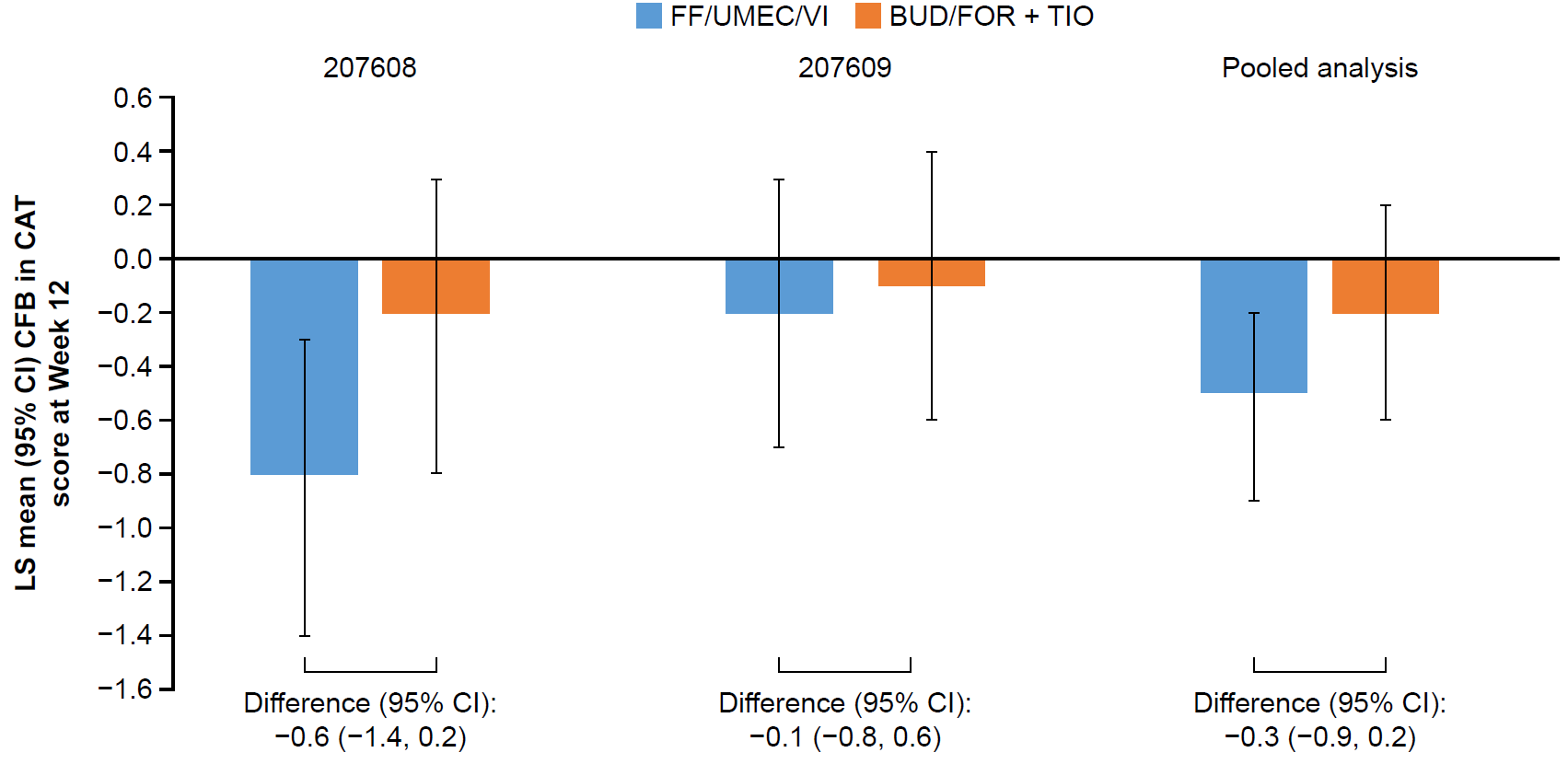


BUD, budesonide; CAT, COPD Assessment Test; CFB, change from baseline; CI, confidence interval; FF, fluticasone furoate; FOR, formoterol; LS, least squares; TIO, tiotropium; UMEC, umeclidinium; VI, vilanterol.

**Supplementary Figure 4.** Change from baseline in weighted mean FEV_1_ at Week 12 by subgroup (pooled ITT population).


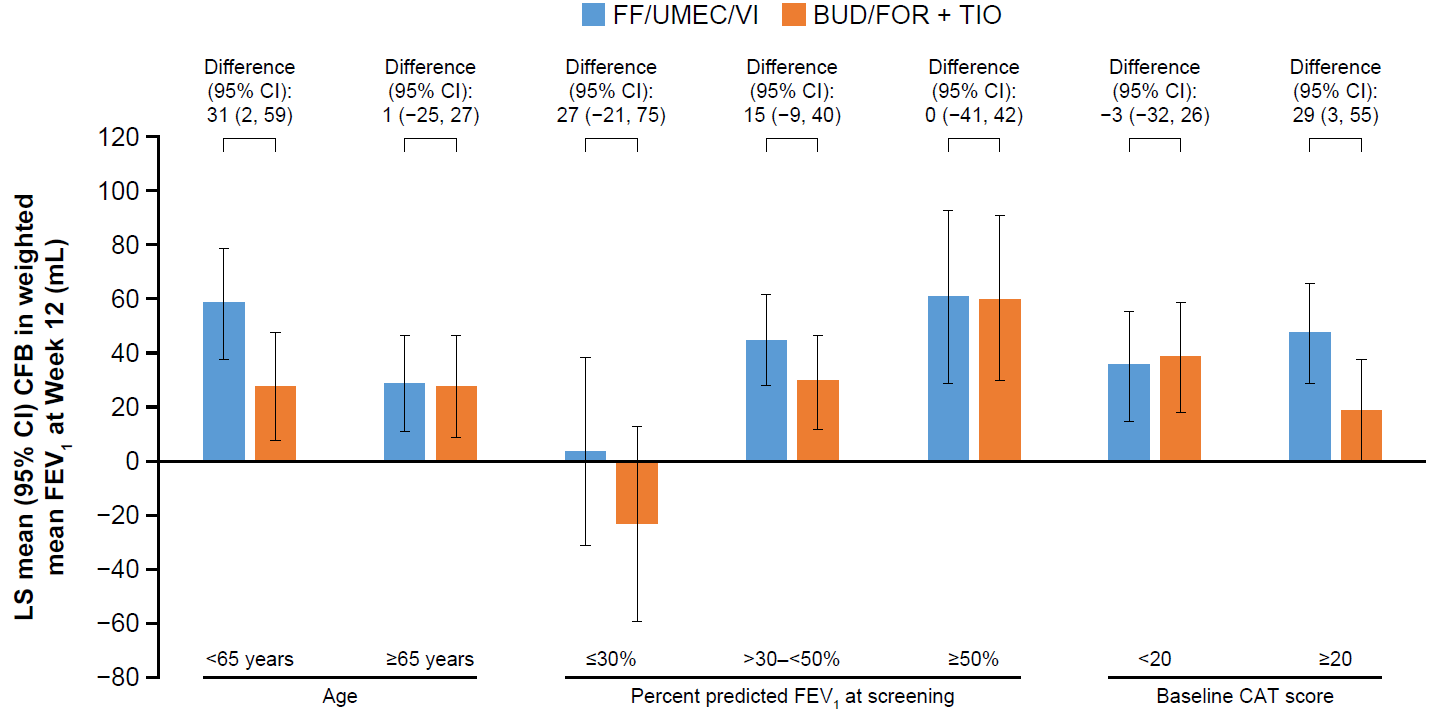


BUD, budesonide; CAT, COPD assessment test; CFB, change from baseline; CI, confidence interval; FEV_1_, forced expiratory volume in 1 second; FF, fluticasone furoate; FOR, formoterol; ITT, intent-to-treat; LS, least squares; TIO, tiotropium; UMEC, umeclidinium; VI, vilanterol.

**Supplementary Figure 5.** Change from baseline in trough FEV_1_ at (A) Day 84 and (B) Day 85 by subgroup (pooled ITT population).


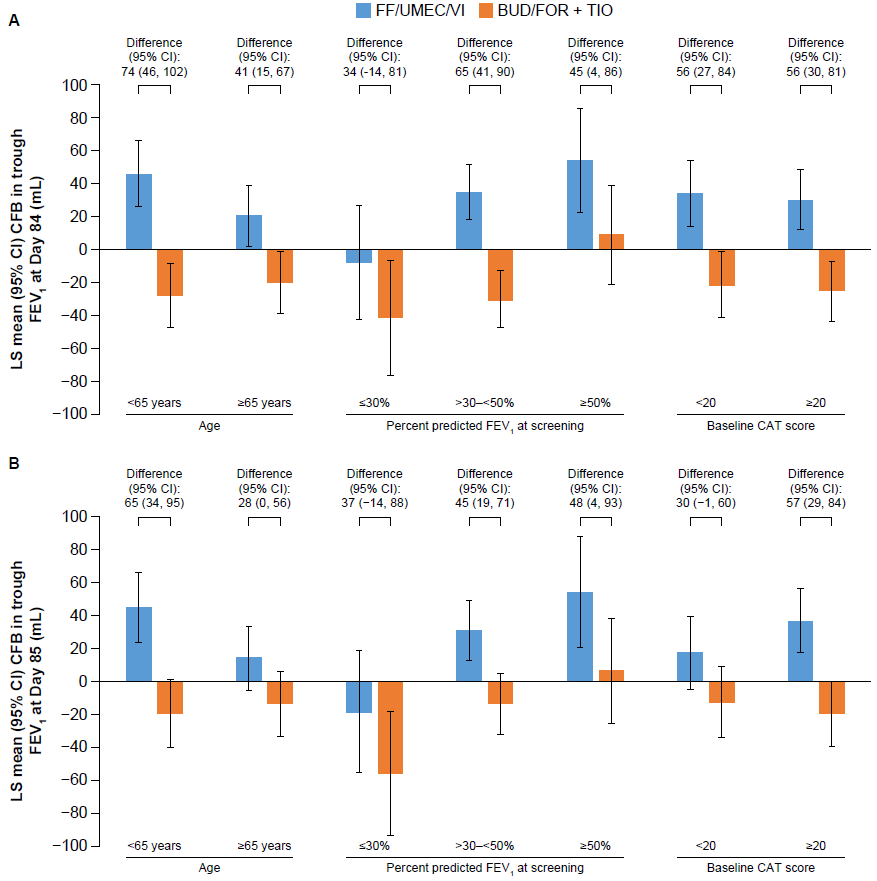


BUD, budesonide; CAT, COPD assessment test; CFB, change from baseline; CI, confidence interval; FEV_1_, forced expiratory volume in 1 second; FF, fluticasone furoate; FOR, formoterol; ITT, intent-to-treat; LS, least squares; mPP, modified per protocol; TIO, tiotropium; UMEC, umeclidinium; VI, vilanterol.
